# Close encounters of three kinds: impacts of leg, wing and body collisions on flight performance in carpenter bees

**DOI:** 10.1242/jeb.245334

**Published:** 2023-05-05

**Authors:** Nicholas P. Burnett, Stacey A. Combes

**Affiliations:** Department of Neurobiology, Physiology, and Behavior, University of California, Davis, CA 95616, USA

**Keywords:** Obstacles, Biomechanics, Hymenoptera, Wind, Maneuverability

## Abstract

Flying insects often forage among cluttered vegetation that forms a series of obstacles in their flight path. Recent studies have focused on behaviors needed to navigate clutter while avoiding all physical contact and, as a result, we know little about flight behaviors that do involve encounters with obstacles. Here, we challenged carpenter bees (*Xylocopa varipuncta*) to fly through narrow gaps in an obstacle course to determine the kinds of obstacle encounters they experience, as well as the consequences for flight performance. We observed three kinds of encounters: leg, body and wing collisions. Wing collisions occurred most frequently (in about 40% of flights, up to 25 times per flight) but these had little effect on flight speed or body orientation. In contrast, body and leg collisions, which each occurred in about 20% of flights (1–2 times per flight), resulted in decreased flight speeds and increased rates of body rotation (yaw). Wing and body collisions, but not leg collisions, were more likely to occur in wind versus still air. Thus, physical encounters with obstacles may be a frequent occurrence for insects flying in some environments, and the immediate effects of these encounters on flight performance depend on the body part involved.

## Teaser

**Summary:** Flying insects frequently collide with obstacles when navigating dense clutter, but the performance consequences of collisions depend on which parts of the insect (e.g. leg, wing, body) are involved.

## INTRODUCTION

Flying animals frequently interact with cluttered vegetation in their habitats. Many birds, for instance, nest and perch in trees and pursue prey through dense foliage (Robinson and Holmes, 1984; Roth et al., 2002), and many insects forage for nectar and pollen among dense patches of flowers (Comba, 1999; Hennessy et al., 2020). In each case, animals navigate around a series of vegetative structures that functionally serve as obstacles and constrain navigable paths (Ravi et al., 2020, 2022). Traversing obstacles while in flight requires coordinated detection of obstacles (e.g. visually) and rapid alteration of the flight path, for example by decelerating, accelerating or changing flight orientation (Baird and Dacke, 2012, 2016; Burnett et al., 2020; Crall et al., 2015; Fabian et al., 2022). Although there are numerous strategies for avoiding obstacles, physical encounters with obstacles (e.g. collisions) can occur frequently: when the bumblebee *Bombus terrestris* traverses gaps that are close in size to its wingspan, contact between the wings and the obstacles and contact between the body and the obstacles each occur in about 40% of flights (Ravi et al., 2020). Similarly, in three species of hawks that are specialized predators in forest habitats, healed fractures in the pelvic girdle – presumably due to collisions with tree branches when pursuing prey in forest canopies – occur in about 19% of individuals (Roth et al., 2002). Despite the prevalence of obstacle encounters in nature, most studies of obstacle traversal in flight focus on behaviors required to avoid obstacles, with little consideration of what happens to organisms when obstacle encounters occur. This contrasts with studies of terrestrial locomotion that consider obstacle encounters as an integral part of traversing terrestrial landscapes (Jayaram and Full, 2016; Jayaram et al., 2018; Wang et al., 2022). Thus, we know relatively little about the effects of physical encounters with obstacles on the performance of flying animals.

Encounters with obstacles can alter locomotion during and soon after the collision and lead to performance-altering injuries that are immediate or cumulative. Intuitively, the effect of obstacle encounters seems likely to involve some ballistic component, i.e. an animal's motion is redirected or slowed. However, observed effects may deviate from intuition based on the animal's mechanics and behavior, and details of the obstacle encounter, such as the initial animal motion and which body structures contact the obstacle (Jayaram et al., 2018). Tolerance for collisions may vary between taxa – for instance, birds of prey can suffer bone fractures that eventually heal, whereas damage from wing collisions in insects is permanent (Foster and Cartar, 2011; Roth et al., 2002). And although numerous kinds of collisions occur in insects – e.g. wing, body and leg collisions in *B. terrestris* (Ravi et al., 2020) *–* wing collisions are the primary focus of many insect studies because cumulative wing damage can contribute to mortality (Crall et al., 2015; Mountcastle and Combes, 2014; Mountcastle et al., 2016; Ravi et al., 2019). Furthermore, numerous insect species have wing morphologies that minimize damage by flexibly deforming during collisions, and these features have become the focus of studies aimed at extracting wing designs for bio-inspired flying vehicles (Combes, 2010; Jankauski et al., 2022; Mountcastle et al., 2019; Phan and Park, 2020). As a result, our knowledge about obstacle encounters in flying insects is heavily focused on a specific anatomical structure, the wing, even though obstacle encounters and injuries can occur to other body parts (Ravi et al., 2020; Roth et al., 2002). We therefore know little about the consequences of obstacle encounters beyond wing collisions that occur during insect flight.

Here, we used the valley carpenter bee, *Xylocopa varipuncta*, to determine the types of obstacle encounters that can occur in flying insects and their consequences for performance. Among bees (family Apidae), carpenter bees in the genus *Xylocopa* are exceptionally large (wing span >4 cm) and are important pollinators of crops and wild plants (Somanathan et al., 2019). Thus, they commonly face the challenge of maneuvering a large body through dense foliage. *Xylocopa* spp. are also models for physiological and neurobiological studies because of their large flight muscles and visual acuity (Roberts et al., 2004; Somanathan et al., 2019). We used high-speed video cameras to film *X. varipuncta* flying through narrow gaps in an obstacle course with varying environmental conditions, including moving versus stationary obstacles and wind versus still air, in a laboratory flight tunnel. Using these data, we answered three questions: (1) how frequent are different obstacle encounters?; (2) what environmental factors affect the likelihood of encounters?; and (3) what are the immediate performance consequences of each encounter?

## MATERIALS AND METHODS

**Fig. 1.**
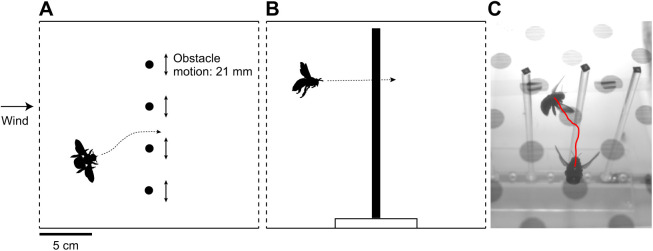
**Schematic diagram of the flight tunnel.** (A) Top and (B) side representations, and (C) oblique camera views of the tunnel used in flight experiments. C shows two super-imposed video frames of a bee traversing stationary obstacles, approximately 400 ms apart. In this flight, there were no obstacle encounters. The red line shows the tracked position of the thorax between the two images. The faded circles were printed on the top of the flight tunnel, as a visual landmark for the bees.

Female carpenter bees, *Xylocopa varipuncta* (Patton 1879) (*n*=15), were collected from the University of California, Davis campus and used immediately for flight experiments. Individual bees were placed in a flight tunnel (20×19×115 cm; width×height×length) (Burnett et al., 2020, 2022), which included a series of vertical obstacles that spanned the middle of the tunnel (obstacle diameter 7 mm, space between obstacles 34.44±2.80 mm, mean±s.d.). A schematic diagram of the tunnel and an example camera view are shown in Fig. 1. The bees' wing spans (45.19±2.11 mm, tip to tip) were larger than the distance between obstacles; thus, bees needed to rotate their body (e.g. yaw) to pass between obstacles. Obstacles were attached to a mechanical arm that oscillated laterally (amplitude 21 mm, frequency 2 Hz) or remained stationary. Fans at each end of the tunnel could be turned on to produce a gentle breeze (mean velocity 0.54 m s^−1^) or off for still air. Wind direction was constant: bees flying in one direction experienced headwinds and in the other direction tailwinds. Up to 12 flights through the obstacles were elicited per bee, using full-spectrum lights at each end of the tunnel (Burnett et al., 2020, 2022). Obstacle motion (stationary versus moving) was fixed for a given bee, but all bees experienced wind and still air, with wind condition switched after approximately six flights and the order of wind conditions alternated between bees. Thus, test conditions were still air with stationary obstacles (*n*=40 flights) or moving obstacles (*n*=34), and wind with stationary obstacles (*n*=42) or moving obstacles (*n*=29).

Flights were filmed at 1500 frames s^−1^ with two synchronized Phantom v611 cameras (Vision Research, Inc., Wayne, NJ, USA) positioned 30 deg from the vertical on opposite sides of the obstacles. Cameras were calibrated using a standard checkerboard calibration method and MATLAB functions (Heikkila and Silven, 1997; Zhang, 2000). In each video, the positions of the bee's head (midpoint between antennae), thorax (approximating the body centroid) and wing tips were tracked with the machine-learning software DeepLabCut (Mathis et al., 2018). Tracked points were checked and manually corrected, and obstacle positions labeled using DLTdv6 in MATLAB (Hedrick, 2008). Labeled positions were converted from 2D coordinates in each camera view into 3D space using MATLAB functions.

**Fig. 2.**
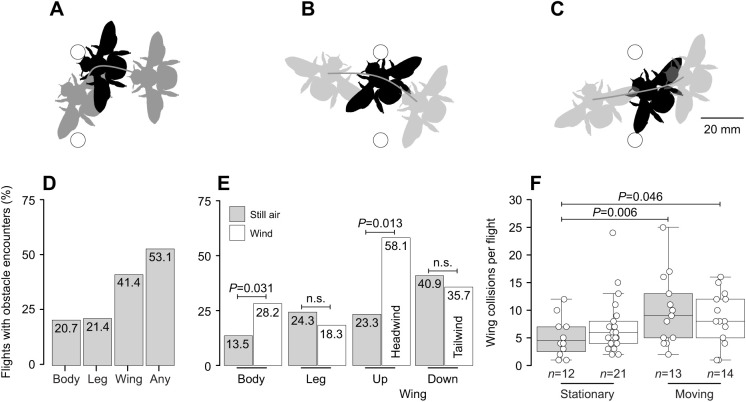
**Types of obstacle encounters observed in carpenter bees.** (A–C) Real examples of (A) body, (B) leg and (C) wing collisions in bees flying past obstacles from right to left (see Movies 1–3). Black shows the moment of each encounter. Gray shows body positions 20 ms before and after encounters. White circles show obstacle positions. (D) Frequency of encounter types, and (E) frequency of encounters grouped by wind (still air or wind) and, for wing collisions, by flight direction. The percentage of each encounter type is indicated within the bars. Up and Down refer to separate directions in the flight tunnel. (F) Number of wing collisions per flight (excluding flights without wing collisions), grouped by wind (still air or wind) and obstacle motion (stationary or moving). Box plots indicate median, upper and lower quartiles, and 1.5 times the interquartile range. Statistical comparisons are indicated by brackets (*P*<0.05 for significance; n.s., not significant). Only environmental factors (i.e. wind, flight direction, obstacle motion) retained by the models are shown.

We classified and counted each obstacle encounter. The most common encounters were body collisions (head, thorax or abdomen contacted obstacles), leg collisions (one or more forelegs contacted obstacles) and wing collisions (the distal half of one or more forewings contacted obstacles) (Fig. 2). Movies 1–3 show examples of each encounter.

To test which experimental conditions contributed to the occurrence of encounters, we used a generalized linear mixed-effects model (GLMM). Models were implemented as logistic regression models with the ‘glmer’ function in the R package *lme4* (https://CRAN.R-project.org/package=lme4), with variables for wind (yes versus no), flight direction (upstream versus downstream) and obstacle motion (moving versus stationary). Bee identity was included as a random effect to account for multiple observations per individual. We allowed for statistical interactions between all experimental variables and generated alternative models by removing terms in a stepwise selection process. Models were compared by their Akaike's information criterion (AIC) via the ‘AIC’ function in the R package *stats* (https://CRAN.R-project.org/package=STAT). Models with the lowest AIC were evaluated with the ‘Anova’ function from the R package *car* (https://CRAN.R-project.org/package=car).

Among flights with obstacle encounters, there was wider variation in the number of wing collisions per flight (range 1–25) compared with the number of body collisions (range 1–2) or leg collisions (maximum of 1). We tested which experimental variables best predicted the number of wing collisions per flight by using a GLMM with a Poisson distribution on flights with at least one wing collision. Model selection and evaluation were carried out as described above. *Post hoc* comparisons of model terms were conducted with Tukey HSD tests using the ‘lsmeans’ function in the R package *emmeans* (https://CRAN.R-project.org/package=emmeans)*.*

We assessed how obstacle encounters affected flight performance. In every video, we identified the first occurrence of each encounter type and defined a 20 ms period before and after each event. This temporal window allowed us to quantify performance immediately before and after encounters, following Mountcastle et al. (2019). Occasionally, pre- and post-encounter periods contained additional collisions, a common outcome when flying near clutter, but the narrow analysis window allowed us to examine changes in flight performance primarily occurring around the focal obstacle encounter. Videos yielded one, two or three encounter types (*n*=42, 26 and 9 flights, respectively).

For each encounter, we measured the change in horizontal ground speed and yaw angle between the pre- and post-encounter periods, as well as the post-encounter yaw rate, where yaw angle was the body angle about the vertical axis. To calculate kinematics, we smoothed 3D position data with cubic smoothing spline curves via the ‘smooth.spline’ function in *stats*. Horizontal ground speed was calculated as the change in *x*–*y* position (lateral and longitudinal movements, omitting vertical motion) per time. Yaw was calculated by converting the Cartesian coordinates of the head and thorax to spherical coordinates via the ‘cart2sph’ function in the R package *pracma* (https://CRAN.R-project.org/package=pracma) and finding the horizontal angle between the body points and the tunnel's long axis. Yaw rate was calculated as change in yaw per time. We considered change in ground speed and yaw, pre- to post-encounter, because initial ground speeds (pre-encounter) were lower for leg collisions (0.18±0.08 m s^−1^, mean±s.d.) than for body collisions (0.26±0.15 m s^−1^) and wing collisions (0.23±0.08 m s^−1^) (Fig. S1). However, initial yaw angles were similar between all encounter types (34.30±24.62 deg; Fig. S1).

We used a linear mixed-effects model to test whether the change in flight metrics depended on encounter type, wind condition and/or obstacle motion. Models were implemented with the ‘lme’ function in the R package *nlme* (https://CRAN.R-project.org/package=nlme). Model selection, evaluation and *post hoc* comparisons were carried out as described above. Assumptions of normality and homogeneity of variances were checked with Shapiro's tests and Levene's tests, respectively. When necessary, variance structures of model terms were modified using the ‘varIdent’ function in *nlme*.

## RESULTS AND DISCUSSION

Of the 145 recorded flights from *X. varipuncta*, 20.7% (*n*=30) included a body collision, 21.4% (*n*=31) included a leg collision and 41.4% (*n*=60) included a wing collision; overall, 53.1% (*n*=68) included some type of obstacle encounter (Fig. 2D). Body collisions were more likely to occur in wind (frequency 28.2%, *n*=20/71 flights) versus still air (13.5%, *n=*10/74 flights) (GLMM: χ^2^=4.673, d.f.=1, *P*=0.031), whereas leg collisions showed similar frequencies between wind (18.3%, *n=*13/71 flights) and still air (24.3%, *n*=18/74 flights; Fig. 2E) (χ^2^=1.426, d.f.=1, *P*=0.232). Wing collision frequency depended on wind and flight direction (χ^2^=6.341, d.f.=1, *P*=0.012), such that flights in headwinds (but not tailwinds) were more likely to contain wing collisions (58.1%, *n=*25/43 flights) than flights in the same direction with still air (23.3%, *n*=7/30 flights; Fig. 2E) (Tukey HSD tests: *P*=0.013). Notably, our AIC-based model selection process indicated that obstacle motion was not a strong predictor of the likelihood of any encounter type.

Among flights with wing collisions, the number of wing collisions per flight depended on wind and obstacle motion (χ^2^=7.011, d.f.=1, *P*=0.008; Fig. 2F). Flights in still air with stationary obstacles had fewer wing collisions (5.3±3.5 wing collisions, mean±s.d.) than flights in still air with moving obstacles (9.9±6.5 wing collisions) (Tukey HSD test: *P*=0.006) or flights in wind with moving obstacles (8.6±4.8 wing collisions) (*P*=0.046).

**Fig. 3.**
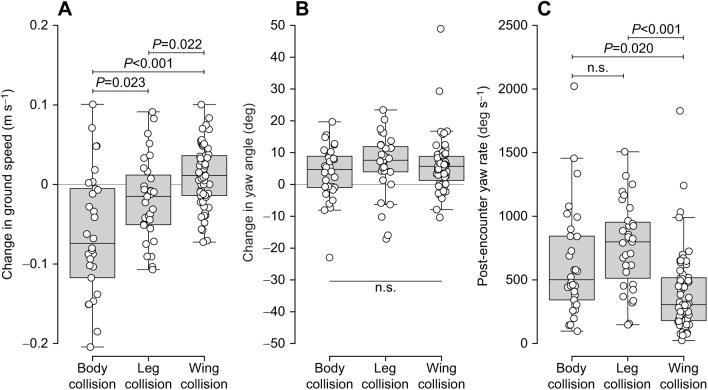
**Effects of obstacle encounters on flight.** Changes in (A) horizontal ground speed and (B) yaw angle between pre- and post-encounter periods, and (C) post-encounter yaw rate (*n*=30 body collisions, 31 leg collisions, 60 wing collisions). Increased yaw (B) indicates rotations away from the tunnel's centerline. Box plots indicate median, upper and lower quartiles, and 1.5 times the interquartile range. Statistical comparisons are indicated by brackets (*P*<0.05 for significance; n.s., not significant).

Body, leg and wing collisions (Fig. 2A–C) each had distinctive effects on ground speed, with large decreases after body collisions (−0.07±0.08 m s^−1^), small decreases after leg collisions (−0.02±0.05 m s^−1^), and small increases after wing collisions (0.01±0.04 m s^−1^) (Fig. 3A) (χ^2^=25.896, d.f.=2, *P*<0.005; Tukey HSD tests: *P*<0.05).

Changes in yaw angle resulting from collisions were not affected by encounter type (χ^2^=0.574, d.f.=2, *P*=0.751) (Fig. 3B) but were affected by wind, with larger changes in yaw angle in still air (7.29±6.12 deg) versus wind (3.96±10.33 deg) (χ^2^=7.378, d.f.=1, *P*=0.007). Post-encounter yaw rate depended on encounter type (χ^2^=21.284, d.f.=2, *P*<0.005) (Fig. 3C), with slower rates after wing collisions [median 305.6 deg s^−1^ (interquartile range, IQR, 333.6 deg s^−1^)] versus body collisions [501.5 deg s^−1^ (IQR 491.4 deg s^−1^)] (Tukey HSD test: *P*=0.020) and leg collisions [799.2 deg s^−1^ (IQR 440.3 deg s^−1^)] (*P*<0.005) (Fig. 3C).

Carpenter bees experienced three types of obstacle encounters when traversing challenging flight environments. Wing collisions were the most frequent encounter (occurring in 41.4% of flights), whereas body and leg collisions each occurred in approximately 20% of flights. The wing collision frequency reported here closely matches the frequency for *B. terrestris* (∼40%) flying through gaps that are geometrically similar to the gaps used in the present study (gap size/wingspan=0.76) (Ravi et al., 2020). However, the body collision frequency for *X. varipuncta* was nearly one-half of the frequency for *B. terrestris* (also ∼40%). Taken together, these results suggest that collisions with obstacles are common for insects flying through natural, complex environments, such as cluttered vegetation. In addition, wing collision frequencies for a range of species may be predicted by general geometrical properties of clutter (i.e. gap size relative to wingspan), whereas other collisions (e.g. body, leg) may depend on species-specific flight strategies and may differ widely between species.

Wind increased the likelihood of some encounters, but this effect was not uniform. Body collisions occurred more frequently in wind, but leg collisions were not affected – and wing collisions occurred more frequently in headwinds. The wind effect may be due to a maneuverability constraint, e.g. bees may be less maneuverable in wind, and therefore less capable of avoiding obstacles (Jakobi et al., 2018). Additionally, wind may prompt shifts in flight strategies as seen in other bees: the honeybee *Apis mellifera* avoids obstacle collisions in still air by changing its flight speed more than changing its flight path but shifts to the opposite strategy in wind (Burnett et al., 2020); when flying towards a target, the bumblebee *Bombus impatiens* uses a multidirectional approach path with smooth deceleration in still air but a unidirectional approach path with little if any deceleration in wind (Chang et al., 2016). Wind-based changes in flight strategy may also account for the larger changes in yaw seen for bees in still air versus wind, although it is unclear whether this outcome is due to an active or passive behavior. Overall, we cannot conclude a specific cause for the unique correlations between wind and obstacle encounters in carpenter bees.

Obstacle motion did not affect the likelihood of any encounter type, although obstacle motion (in still air or wind) led to a greater number of wing collisions for flights in which wing collisions occurred (Fig. 2F). Thus, the number of wing collisions per flight (and amount of wing damage inflicted) is not strictly a function of flapping frequency and flight speed, e.g. the number of up and down wing strokes while the bee is within one wing length of the obstacle. In particular, the number of wing collisions per flight may be influenced by obstacle motion and oblique flight paths that extend the amount of time that the bee is near the obstacle, as well as physical feedback between the wing and the obstacle that rotates the bee (by the wing acting as a moment arm) or halts the bee's translational motion (Jankauski et al., 2022; Mountcastle et al., 2019; Phan and Park, 2020). These complex interactions may occur alone or in combination, and either exacerbate or minimize the number of subsequent collisions and total amount of wing damage experienced. Given that our experiment tested only one obstacle arrangement, one type of obstacle motion and one mild wind speed, additional studies would improve our understanding of how environmental variables and flight strategies interact to affect the frequency of obstacle encounters within and across flights.

Body, leg and wing collisions each had significant, but unique, impacts on flight performance. Body collisions resulted in the largest change in ground speed (a decrease, on average) with a moderate degree of body rotation, suggesting a large portion of the bee's translational kinetic energy was lost during the collision (likely transferred to the obstacle) or converted into rotational energy (Fig. 3). Leg collisions, in contrast, resulted in slightly decreased speeds but the most rapid rotations. Wing collisions resulted in small increases in speed and low rotation rates – this result might be caused by bees accelerating, on average, past the obstacles and the flexible wings striking and deforming against the obstacles with little effect on the bee. Collisions with stiff wings would transfer forces to the body, causing body rotations and decreased ground speeds (Mountcastle et al., 2019). The range of rotation rates for body, leg and wing collisions in *X. varipuncta* (up to ∼2000 deg s^−1^) closely matches the range of rotation rates for wing collisions in an insect-inspired, micro-aerial vehicle (MAV) with flexible wings, described by Mountcastle et al. (2019). However, median rotation rates for body, leg and wing collisions in *X. varipuncta* (501.5, 799.2 and 305.6 deg s^−1^, respectively) were each lower than the median rotation rates for the MAV's wing collisions, 1205 deg s^−1^, possibly because the MAV had a higher flapping frequency (140 versus ∼120 Hz) and smaller mass (80 versus 400–1000 mg) than *X. varipuncta* (Chirarattananon et al., 2014; Roberts et al., 2004). Overall, these differences suggest that complex wing anatomies, e.g. continuous gradients in flexibility (Combes and Daniel, 2003), and movements of real insects provide better damping of post-collision body rotations than what is known for insect-inspired MAVs, although extreme body rotations can occur in both.

The relative stability of carpenter bees following wing collisions is not surprising given that flexible insect wings are already sources of bio-inspiration for MAVs, where stability after wing collisions is desired (Jankauski et al., 2022; Mountcastle et al., 2019; Phan and Park, 2020). However, our results suggest that body and leg collisions could also serve as sources of bio-inspiration, either for their mechanical design to withstand impacts or through their use when traversing narrow gaps. For instance, rapid decreases in ground speeds and simultaneous rotations seen during body collisions could inspire collision-resistant body designs that help execute rapid turns (Jayaram et al., 2018). Furthermore, leg collisions achieved the most extreme rapid body rotations (Fig. 3B,C), suggesting that intentional leg (or other appendage) contact with obstacles may allow MAVs to quickly and accurately navigate clutter without relying on changes to the magnitude or relative direction of aerodynamic force production.

Our study confirms that diverse obstacle encounters occur for insects navigating clutter, but we also show that these encounters occur with different frequencies, are uniquely shaped by environmental factors (e.g. wind), and have unique consequences for flight performance. While most insect flight studies focus on collision-free flights, our study highlights that physical encounters with obstacles are frequent in cluttered habitats and that their effects should be considered as an integral component of navigating challenging areas. Shifting our focus from collision avoidance during flight to the full spectrum of flight behaviors and body designs that withstand and utilize collisions to regulate flight speed and body orientation is a rich area of research for both biologists and engineers. Future studies should investigate additional aspects of obstacle encounters, including characteristics of obstacles (e.g. size, shape, stiffness, spacing), how they vary between insect species or across an individual's lifetime, and whether obstacle encounters can be controlled to enhance flight performance and maneuverability of insects in clutter.

## Supplementary Material

10.1242/jexbio.245334_sup1 Supplementary information
