# Supplementary material for: Close encounters of three kinds: impacts of leg, wing and body collisions on flight performance in carpenter bees
Source: J Exp Biol. 2023 May 5;226(9):jeb245334. doi: 10.1242/jeb.245334 (PMC10184765; doi:10.1242/jeb.245334)
Supplement: Supplementary information [file jexbio-226-245334-s1.pdf]

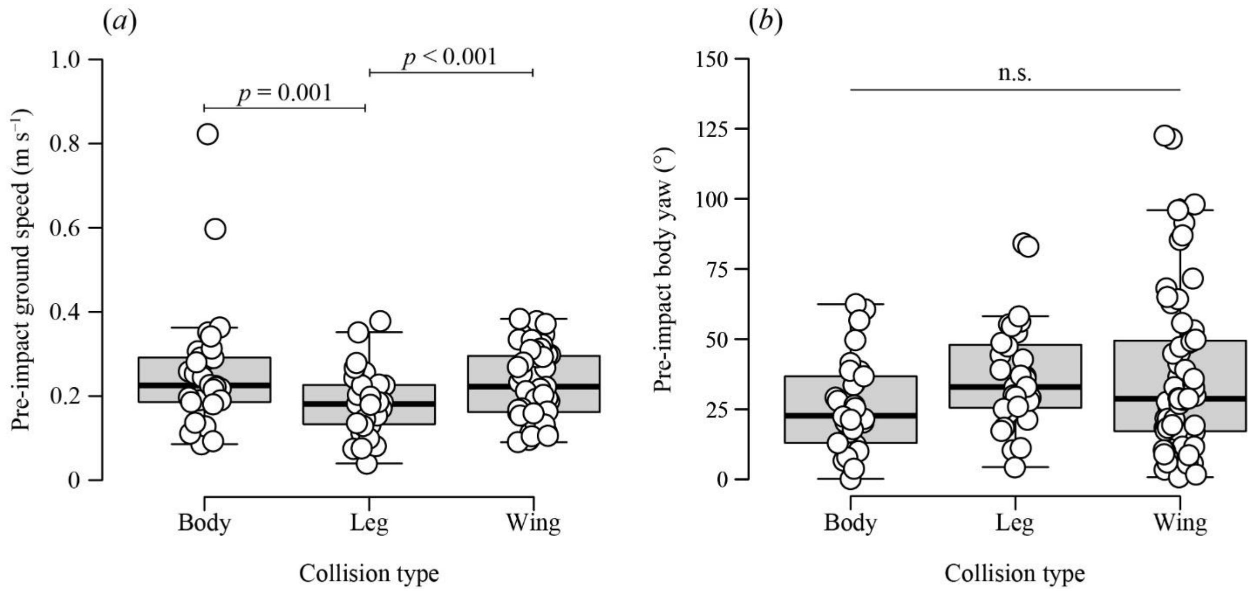

Fig. S1. Initial flight performance of bees prior to obstacle encounters. (*a*) Pre-impact ground speeds and (*b*) body angles, calculated over the 20 ms before each encounter. Brackets show statistical comparisons ($p < 0.05$ for significance; 'n.s.' = not significant). Statistical comparisons and model selections were done as described in the main text.

Journal of Experimental Biology • Supplementary information

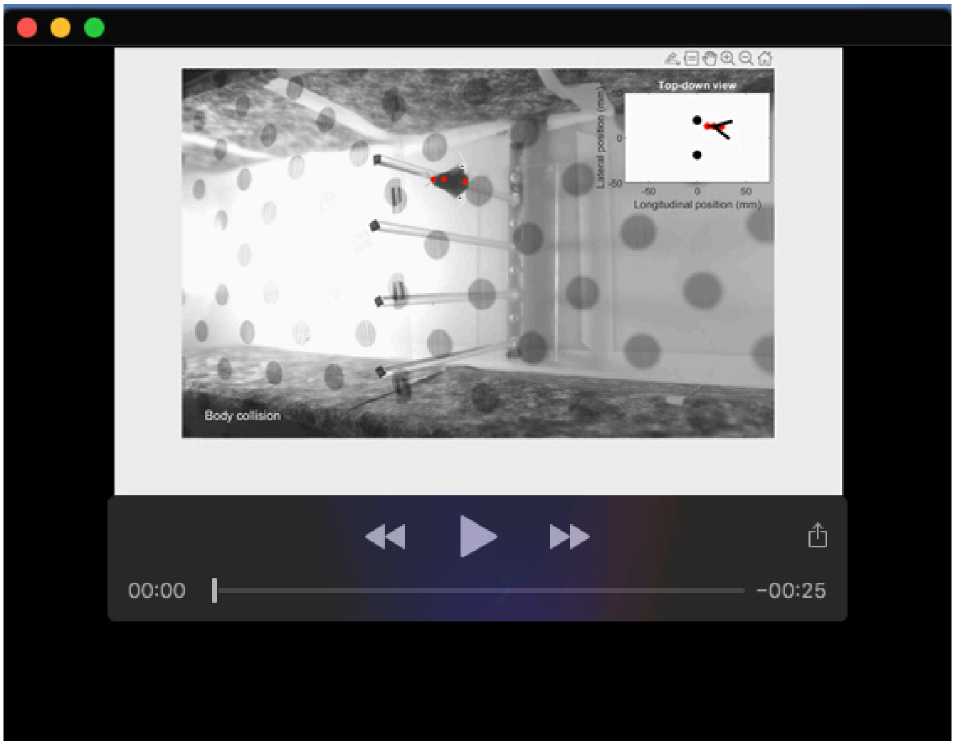

Movie 1. Example video of a body collision.

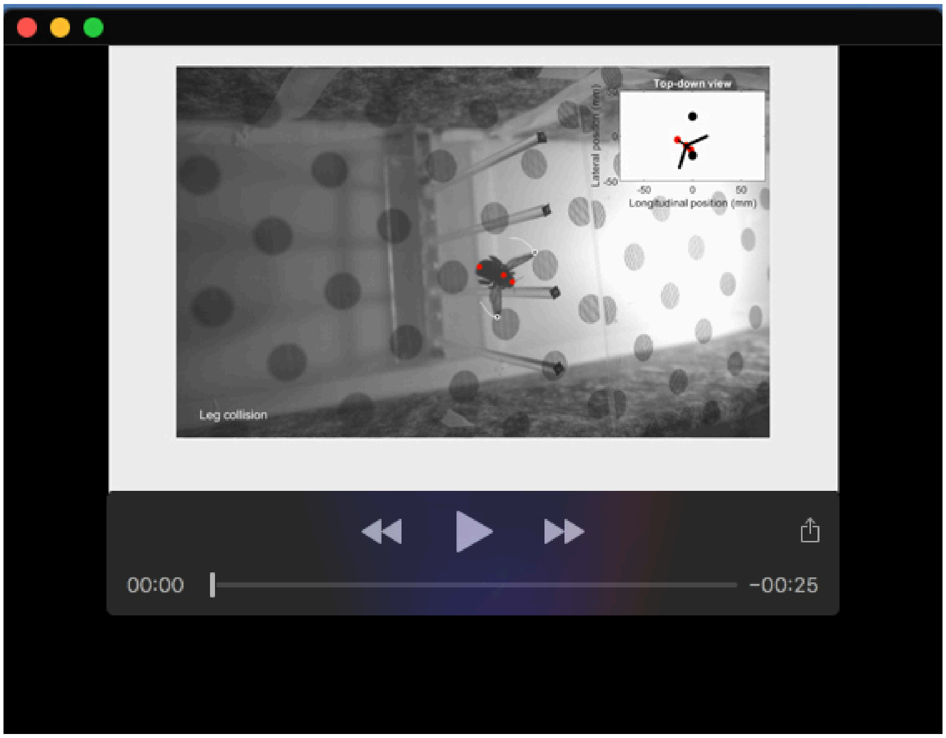

Movie 2. Example video of a leg collision.

Journal of Experimental Biology • Supplementary information

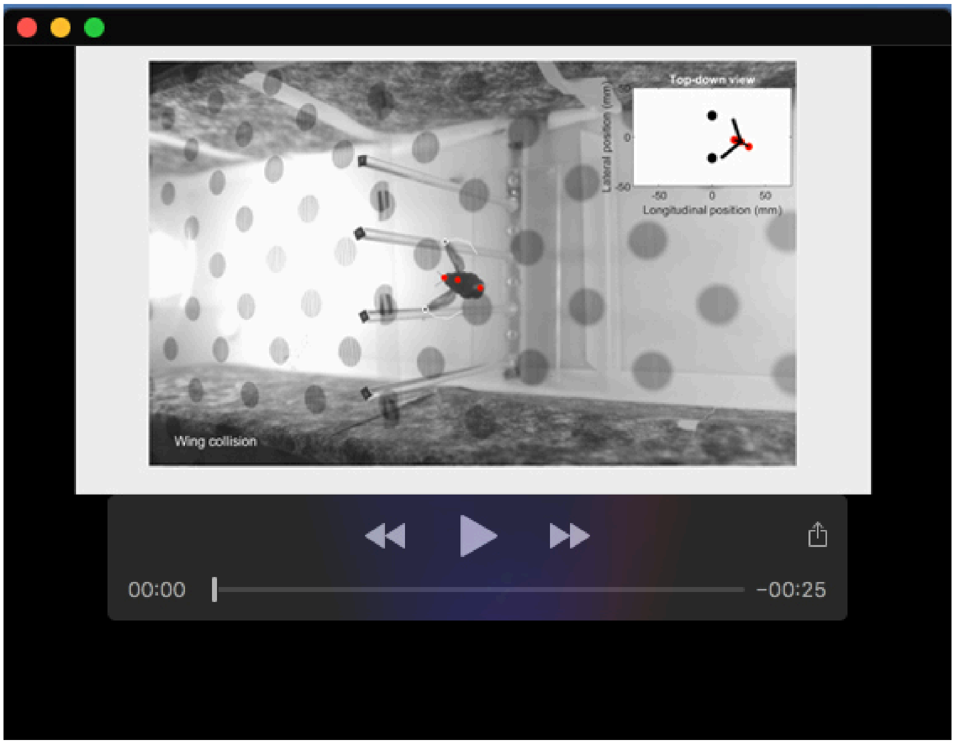

Movie 3. Example video of a wing collision.

Journal of Experimental Biology • Supplementary information
